# No Effect of Thyroid Dysfunction and Autoimmunity on Health-Related Quality of Life and Mental Health in Children and Adolescents: Results From a Nationwide Cross-Sectional Study

**DOI:** 10.3389/fendo.2020.00454

**Published:** 2020-09-02

**Authors:** Raphael Hirtz, Anne Keesen, Heike Hölling, Berthold P. Hauffa, Anke Hinney, Corinna Grasemann

**Affiliations:** ^1^Division of Pediatric Endocrinology and Diabetology, Department of Pediatrics II, University Hospital Essen, University of Duisburg-Essen, Essen, Germany; ^2^Department of Child and Adolescent Psychiatry and Psychotherapy, University Hospital Essen, University of Duisburg-Essen, Essen, Germany; ^3^Department of Epidemiology and Health Monitoring, Robert Koch Institute, Berlin, Germany; ^4^Department of Pediatrics and Center for Rare Diseases Ruhr CeSER, St. Josef-Hospital, Ruhr University Bochum, Bochum, Germany

**Keywords:** health-related quality of life, mental health, SDQ, subclinical hypothyroidism, subclinical hyperthyroidism, overt hypothyroidism, overt hyperthyroidism, autoimmune thyroiditis

## Abstract

**Background:** In adults, a significant impact of thyroid dysfunction and autoimmunity on health-related quality of life (HRQoL) and mental health is described. However, studies in children and adolescents are sparse, underpowered, and findings are ambiguous.

**Methods:** Data from 759 German children and adolescents affected by thyroid disease [subclinical hypothyroidism: 331; subclinical hyperthyroidism: 276; overt hypothyroidism: 20; overt hyperthyroidism: 28; Hashimoto's thyroiditis (HT): 68; thyroid-peroxidase antibody (TPO)-AB positivity without apparent thyroid dysfunction: 61] and 7,293 healthy controls from a nationwide cross-sectional study (“The German Health Interview and Examination Survey for Children and Adolescents”) were available. Self-assessed HRQoL (KINDL-R) and mental health (SDQ) were compared for each subgroup with healthy controls by analysis of covariance considering questionnaire-specific confounding factors. Thyroid parameters (TSH, fT4, fT3, TPO-AB levels, thyroid volume as well as urinary iodine excretion) were correlated with KINDL-R and SDQ scores employing multiple regression, likewise accounting for confounding factors.

**Results:** The subsample of participants affected by overt hypothyroidism evidenced impaired mental health in comparison to healthy controls, but SDQ scores were within the normal range of normative data. Moreover, in no other subgroup, HRQoL or mental health were affected by thyroid disorders. Also, there was neither a significant relationship between any single biochemical parameter of thyroid function and HRQoL or mental health, nor did the combined thyroid parameters account for a significant proportion of variance in either outcome measure. Importantly, the present study was sufficiently powered to identify even small effects in children and adolescents affected by HT, subclinical hypothyroidism, and hyperthyroidism.

**Conclusions:** In contrast to findings in adults, and especially in HT, there was no significant impairment of HRQoL or mental health in children and adolescents from the general pediatric population affected by thyroid disease. Moreover, mechanisms proposed to explain impaired mental health in thyroid dysfunction in adults do not pertain to children and adolescents in the present study.

## Introduction

Up to 30 percent of adult patients affected by thyroid dysfunction have been reported to suffer from psychological impairments despite proper medical treatment (1), and up to 20 percent are affected by depression (2). Whether children and adolescents with thyroid disease are likewise affected by impaired health-related quality of life (HRQoL) or mental health has not yet been rigorously investigated, despite thyroid disorders being a common endocrine disease in this age group. Aberrant laboratory findings of the hypothalamus-pituitary-thyroid axis are detected in about 3.5% of children and adolescents on occasional testing (3), and thyroid disorders are among the top 5 reasons for referral to pediatric endocrinology (4).

Subclinical hypothyroidism (HYPO_SC_), defined by an elevated thyrotropin (TSH) level above the age-specific reference range despite a normal free T4 (fT4), is a common finding in children and adolescents with an estimated prevalence of 1.7 to 2.9% (5, 6). Studies investigating HRQoL and mental health in pediatric HYPO_SC_ are rare, and findings are ambiguous. While Holtmann et al. (7) found significantly higher TSH levels in children and adolescents affected by impaired mental health, neither Cerbone et al. (8) nor Zepf et al. (9) could relate HYPO_SC_ to affective and behavioral dysregulation. However, pediatric sample sizes so far have been small. Considering only subtle effects of HYPO_SC_ on clinical and metabolic outcomes (5), a larger sample size might be necessary to disclose effects of HYPO_SC_ on HRQoL and mental health in children and adolescents.

In overt hypothyroidism (HYPO_OVERT_), there is a significantly elevated TSH accompanied by a fT4 level below the age-specific reference range. The most common cause of HYPO_OVERT_ is Hashimoto's thyroiditis (HT) (5), a condition that is the most common autoimmune disease in children and adolescents (10, 11). In adults, several studies found an impaired HRQoL in HT [e.g. (12–16)], even in patients treated with levothyroxine (1, 17). Notably, most of the studies reported a significant linear and inverse relationship between TPO-AB levels and HRQoL (14, 17–19). Data regarding the relationship between HT and HRQoL or mental health in children and adolescents are rare. A single study focusing on adolescent patients with type 1 diabetes showed an impaired HRQoL in those patients with levothyroxine treated HT (20). However, no research investigating the relationship between mental health and HT has been conducted even though HT and hypothyroidism have been related to depression and anxiety disorders in adults by a recent large-scale meta-analysis (21).

In recent years, improved TPO-AB assays (22) and the resampling of reference populations for assay calibration (23) have resulted in lower cut-off levels for TPO-AB positivity. Also, there is a growing awareness of an increase in TPO-AB positivity with the onset of puberty, especially in girls (24–26). However, the clinical phenotype, including HRQoL and mental health, of children and adolescents who only evidence increased TPO-AB levels but no signs of thyroid dysfunction (i.e., normal thyroid hormone levels, unremarkable ultrasonography of the thyroid gland; subsequently TPO_only_) is not well-characterized and, thus, information on the relevance of this condition is lacking.

Subclinical hyperthyroidism (HYPER_SC_), defined in the literature by a TSH level below the age-specific reference range and a normal fT4 [e.g., (27, 28)], is observed in 2.3% of adolescents aged 13–16 years (29). In adults, there is ambiguous evidence regarding the relationship between HYPER_SC_ and HRQoL as well as mental health. While Biondi et al. (30) found an impaired HRQoL in HYPER_SC_ and Kvetny et al. (31) could show a slightly increased risk of subclinical depression, mood and cognition were not affected by HYPER_SC_ in 3 population-based studies (27, 32, 33). No study has been conducted in children and adolescents so far.

HYPER_OVERT_, characterized by a TSH level below and an fT4 above the age-specific reference range, is a rare condition observed in 0.1 to 3 cases in 100,000 children and most commonly caused by Graves' disease (34, 35). In adults, HRQoL is significantly reduced before (36) but also after treatment of Graves' disease (32, 37, 38), independent of the treatment modality. Abraham-Nordling et al. (32) and Riguetto et al. (38) also investigated the relationship between thyroid hormone status and HRQoL, but there was no consistent relationship across studies. Zader et al. (39) recently published findings on the prevalence of mental health disorders in children and adolescents with physician-diagnosed HYPER_OVERT_ and reported a significantly increased risk of a diagnosis of ADHD, adjustment disorder, anxiety, and bipolar disorder as well as depression.

Due to the prevalence and therefore also importance of thyroid dysfunction and thyroid autoimmunity, the present study was intended to investigate the relationship between thyroid disorders and HRQoL as well as mental health in children and adolescents relying on data from a large nationwide cross-sectional study.

Considering the current literature, we tested the following hypotheses:

H_1_: In HT, HRQoL is impaired, and the risk for mental health problems is increased.

H_2_: In HT, TPO-AB levels and HRQoL show a negative correlation.

H_3_: In HYPO_SC_, HRQoL is not affected, and the risk of mental health problems is not increased.

H_4_: In HYPO_SC_, there is no correlation between HRQoL with thyroid hormone status and other markers of thyroid function (including TPO-AB levels and thyroid volume).

H_5:_ In HYPO_OVERT_, HRQoL is impaired, and the risk of mental health problems is increased.

H_6_: In HYPER_OVERT_, HRQoL is impaired, and the risk of mental health problems is increased.

## Materials and Methods

### Study Population

‘The German Health Interview and Examination Survey for Children and Adolescents’ (KiGGS) was conducted by the Robert Koch Institute (RKI) between 2003 and 2006 to provide information on the health status of German children and adolescents. Details on the study design, the sampling strategy, and the study protocol have been described in detail elsewhere (40). Briefly, a two-stage random, clustered, and representative sample of German children and adolescents was selected (40). The final sample included 17,641 children and adolescents (8,656 girls, 8,985 boys; 0–6 years: 6,680, 7–13 years: 7,224, 14–17 years: 3,734). Parents and children aged 11 years and older completed self-administered, standardized questionnaires, and parents additionally participated in a computer-assisted personal interview conducted by a specially trained study physician that also performed a physical examination, thyroid ultrasonography, and phlebotomy for laboratory assessment (40). The study was approved by the Charité Berlin ethics committee as well as the Federal Office for the Protection of Data (40). Written informed consent was obtained from parents as well as from children aged 14 years and older.

Only children and adolescents with information on thyroid function, thyroid autoimmunity, the intake of thyroid medication, and pre-existing thyroid disease as well as HRQoL and mental health (SDQ) were included for analyses. Thus, in total, the sample comprised 8,052 children and adolescents (3,902 girls, 4,150 boys; 3–5 years: 92, 6–12 years: 4,492, 13–18 years: 3,466).

### Questionnaires

The SDQ is a well-established, multi-informant questionnaire with verified reliability and validity conceptualized to screen for mental health symptoms as well as positive attitudes in children and adolescents assessing 5 dimensions (emotional symptoms, conduct problems, hyperactivity/inattention, peer relationship problems, and prosocial behavior) by 25 items on a 3-point Likert scale (0–2). By summing the subscores from each dimension, a total difficulties score (subsequently SDQ-TD score) can be calculated with higher scores indicating more problems (for information on the normal range of SDQ-TD and KINDL-R scores s. Table 1B) (41).

**Table 1A:** Subsample characteristics - demographics.

	**Controls (*N =* 7293)**	**HT (*N =* 68)**	**TPO_**only**_ (*N =* 59)**	**Subclinical hypothyroidism (*N =* 331)**	**Subclinical hyperthyroidism (*N =* 276)**	**Overt hypothyroidism (*N =* 20)**	**Overt hyperthyroidism (*N =* 28)**
Age	12.15 (3.35)	14.16 (2.47)^*^	13.73 (3.32)^*^	11.02 (4.20)^*^	11.00 (4.20)^*^	10.54 (2.89)	12.16 (4.22)
z-BMI	−0.03 (0.98)	0.12 (1.03)	0.16 (0.86)	0.11 (1.07)	−0.11 (0.98)	0.11 (1.04)	−0.32 (0.99)
Gender (female)	48.1%	77.9%^*^	72.9%^*^	46.2%	48.2%	45%	60.7%
TPO positivity	0%^#^	100%^#^	100%^#^	7.3%^*^	1.3%^*^	27.8%^*^	12.5%^*^
Levothyroxine	0%^#^	19.1%^*^	0%^#^^§^	1.5%^*^	0.4%	0%	14.3%^*^
Iodine	0%^#^	8.8%^*^	0%^#^	2.1%^*^	0.4%	0%	3,6%^*^
Diagnosed thyroid disease	0%^#^	29.4%^*^	0%^#^^§^	2.5%^*^	2.3%^*^	5%^*^	21.4%^*^
Altered echogenicity	0%^#^	32.8%	0%^#^	0.9%^+^	0%^+^	5.3%^+^	3.6%^+^
Irregularities US	0%^#^	35.3%^*^	6.8%^*^^§^	2.4%^*^	1.4%^*^	15%^*^	7.1%^*^
Psychotropic medication°	1.4%	0%	1.8%	0.6%	1.9%	5%	0%
General health^$^	86.0%	85.2%	75.0%	85.9%	86.4%	71.4%	86.7%

*Mean and standard deviation in brackets or percentage*.

* *Significant differences in comparison to healthy controls FDR-corrected at q < 0.05*.

§ *Significant mean difference in comparison to HT FDR-corrected at q < 0.05 (also compare to Table S3)*.

# By definition,

+ *not statistically evaluated additionally to irregularities on ultrasonography, ^§^good or very good (parent-rated). °Does not include antiepileptics or phytopharmaceuticals. Note, irregularities on ultrasonography include altered echogenicity. US = ultrasonography*.

**Table 1B:** Subsample characteristics – mental health and thyroid functioning.

	**Controls (*N =* 7293)**	**HT (*N =* 68)**	**TPO_**only**_ (*N =* 59)**	**Subclinical hypothyroidism (*N =* 331)**	**Subclinical hyperthyroidism (*N =* 276)**	**Overt hypothyroidism (*N =* 20)**	**Overt hyperthyroidism (*N =* 28)**
KINDL^$^	75.33 (10.16) (*n =* 7171)	73.93 (9.16) (*n =* 66)	70.98 (9.00) (*n =* 58)	76.78 (9.87) (*n =* 327)	76.55 (10.53) (*n =* 267)	72.74 (7.65) (*n =* 20)	73.61 (10.76) (*n =* 27)
SDQ^$^	9.25 (4.83) (*n =* 7106)	9.30 (4.17) (*n =* 66)	10.56 (4.72) (*n =* 57)	8.80 (4.73) (*n =* 323)	9.70 (4.80) (*n =* 262)	11.42^*^ (4.55) (*n =* 20)	9.22 (4.79) (*n =* 27)
z-TSH	−0.01 (0.86) (*n =* 7293)	1.36^*^ (1.42) (*n =* 68)	0.42^*^^§^ (0.82) (*n =* 59)	2.43^#^ (0.49) (*n =* 331)	−2.59^#^ (0.66) (*n =* 276)	4.18^#^ (3.51) (*n =* 20)	−3.74^*^ (2.39) (*n =* 29)
z-fT4	0.02 (0.85) (*n =* 7293)	−0.01 (1.36) (*n =* 68)	−0.07 (0.90) (*n =* 59)	0.02 (0.90) (*n =* 331)	0.15 (0.88) (*n =* 276)	−3.37^#^ (2.68) (*n =* 20)	3.28^*^ (1.65) (*n =* 28)
z-fT3	0.03 (0.98) (*n =* 7290)	−0.04 (1.11) (*n =* 68)	0.28 (1.01) (*n =* 59)	0.33^*^ (1.10) (*n =* 331)	0.36^*^ (1.667) (*n =* 276)	−0.92^*^ (1.51) (*n =* 20)	2.19^*^ (2.94) (*n =* 28)
TPO-AB (IU/ml)	50.21 (13.86) (*n =* 134)^+^	441.01^*^ (380.39) (*n =* 68)	251.80^*^^§^ (188.00) (*n =* 59)	264.69^#^ (301.92) (*n =* 30)	228.10^#^ (257.00) (*n =* 5)	546.81^#^ (534.84) (*n =* 7)	511.00^#^ (323.36) (*n =* 3)
Urinary iodine excretion (mg/l)	125.55 (77.68) (*n =* 6394)	129.58 (95.73) (*n =* 56)	126.96 (89.88) (*n =* 51)	141.81^*^ (91.78) (*n =* 294)	145.10^*^ (85.30) (*n =* 236)	138.08 (78.25) (*n =* 15)	143.49 (104.58) (*n =* 24)
z-thyroid volume	−0.01 (0.86) (*n =* 7271)	1.83^*^ (1.62) (*n =* 68)	0.66^*^^§^ (0.69) (*n =* 55)	−0.11^*^ (1.20) (*n =* 279)	0.11 (1.09) (*n =* 231)	0.06 (1.50) (*n =* 18)	0.60^*^ (1.21) (*n =* 25)

*Mean, standard deviation as well as the number of cases considered in brackets*.

* *Significant mean differences in contrast to controls FDR-corrected at q < 0.05, also compare to Table 3*.

§ *Significant mean difference in comparison to HT FDR-corrected at q < 0.05, also compare to Table S3*.

# *Not tested*.

+ *Note: Only 134 controls had a TPO-AB titer above the detection threshold of the assay*.

$ *Normal range SDQ-TD scores: <11 years: <16, > 11 years: <17 (42, 43), normal range KINDL-R: ♂ <11 years: > 68, 11–13 years: > 64, 14–18 years: > 59; ♀ <11 years: > 68, 11–13 years: > 63, 14–18 years: > 55 (44)*.

HRQoL was measured using the KINDL-R. The KINDL-R is a widely used either self- or parent-reported questionnaire with good psychometric properties and consists of 24 items assessing 6 dimensions of HRQoL (physical well-being, emotional well-being, self-esteem, family, friends, everyday functioning) scored on a five-point Likert scale (1–5). A total score can be calculated and transformed to values between 0 and 100. Higher scores indicate a better quality of life (44).

While in children younger than 11 years we used the scores on the parent-proxy form of self-administered questionnaires, in children older than 11 years we referred to the scores of the self-report form of the questionnaires for analyses (45).

The computer-assisted interview covered the medical history of the participating children and adolescents and asked parents for selected, previously physician-diagnosed chronic conditions as well as other chronic conditions, including thyroid disease, in an open-ended question format. Moreover, the computer-assisted interview comprised a detailed section on the use of medication within the last 7 days, either prescribed or sold as an over-the-counter drug. To verify the reported medication, parents were asked to bring the original containers or package inserts on the day of the interview. Specific ATC (Anatomical Therapeutic Chemical) codes were recorded for all reported medication (40).

Additional questionnaires covered information on the educational and professional status as well as on the total household income of the participants' families as well as comprehensive information on the migration background of participants, including nationality (40).

### Laboratory Studies

Blood samples were obtained by a venous puncture after a median fasting period of 2 h using a vacutainer system. Whole blood was stored at 4°C and serum at −40°C before the transfer of samples to the central laboratory of the RKI within 3 days after sample collection (46).

Analyses of serum TSH and fT4 levels were performed with the Elecsys 2010® immunoassay analyzer (Roche Professional Diagnostics, Rotkreuz, Germany). TSH and fT4 assays employed are sandwich electrochemiluminescence immunoassays (ECLIA) with an inter-assay variation of <3.9% and <5.3% and a detection range of 0.005 to 100 IU/ml and 0.023 to 7.77 ng/ml, respectively (46). TPO-antibody levels were measured on a Phadia 250® immunoassay system (Thermo Fisher Scientific, Uppsala, Sweden) in conjunction with the ImmunoCap TPO assay, a fluoro-enzyme-immunoassay (FEIA) with an inter-assay variation of <4.9%. According to the package insert, the measurement range of the assay lies between 33.4 and 3600 IU/ml, and the cut-off level for TPO-positivity has been established at > 100 IU/ml.

TSH, fT4, and fT3 levels were z-transformed according to age- and gender-specific reference ranges (for details, please refer to the Supplementary Material).

Urinary iodine excretion was determined from spot urine samples according to the Sandell-Kolthoff reaction as recommended by the WHO. Measurements were performed on a Miras Cobas Plus analyzer [Roche Professional Diagnostics, Rotkreuz, Germany; for a more detailed description of the methodology, please refer to (47)].

### Physical Examination

In children 2 years and older, height was determined in upright posture using a calibrated stadiometer without wearing shoes. Height was recorded with a precision of 0.1 cm. Weight was measured wearing underwear by an electronic scale displaying weight with a precision of 0.1 kg. BMI was determined by the ratio of weight in kg and the height in meters squared (kg/m^2^) (48).

### Ultrasonography

In children 6 years and older, thyroid volume was measured by ultrasonography. The length (*l*), width (*w*), and depth (*d*) of each lobe was measured in transverse and longitudinal views by a 7.5 MHz linear-array transducer in supine position. The volume of each lobe was calculated according to Brunn et al. (49) by multiplying the obtained thyroid dimensions considering a correction factor (*l* × *w* × *d* × 0.479). Total thyroid volume was determined by summing the volume of both lobes. Irregular echogenicity, cysts, nodules as well as uncommonly located thyroid tissue were recorded. Before the study, all investigators were trained on performing a standardized volumetry of the thyroid gland. Throughout the study, average measurements of thyroid volume were compared between investigators to identify and correct systematic errors of measurement [(47); for details on the construction of percentile curves for thyroid volume, please see the Supplemental Material].

### Core Definitions

HYPO_SC_ and HYPER_SC_ were defined by z-standardized TSH levels above and below 1.96 SDS (97.5 percentile), respectively, and z-standardized fT4 and fT3 levels within normal range (±1.96 SDS). In HYPO_OVERT_ and HYPER_OVERT_, z-standardized fT4 or fT3 levels were additionally required to be above and below 1.96 SDS, respectively.

A diagnosis of HT was assumed in participants of the KiGGS study with a TPO-AB level above 100 IU/ml as well as no diagnosis of Graves' disease and at least one of the following conditions: HYPO_SC_ or HYPO_OVERT_, irregularities of echogenicity on thyroid ultrasonography, an increased or decreased thyroid volume, prescription of thyroid medication (levothyroxine), or a previously physician-diagnosed HT. TPO_only_ participants were characterized by elevated TPO-AB levels but no evidence of thyroid dysfunction (normal z-standardized TSH, fT4, fT3 levels, normal thyroid volume and echogenicity, no previously physician-diagnosed thyroid disease, no prescription of thyroid medication). Note, these definitions of HT and TPO_only_ excluded that an euthyroid participant of the KiGGS study with a previously physician-diagnosed HT was misclassified as TPO_only_.

Participants of the KiGGS study without a previously diagnosed thyroid disease, without thyroid medication (levothyroxine, iodine), without elevated TPO-AB levels above the assay cut-off as well as with normal thyroid hormone levels (TSH and fT4) and normal thyroid volume on ultrasonography were classified as healthy controls. According to this definition, patients with congenital hypothyroidism and thyroidectomy were either excluded from the group of healthy controls or only considered when on inadequate levothyroxine replacement therapy.

### Statistics—Overview

All analyses, including *post-hoc* contrasts, if applicable, without hypotheses, were tested two-tailed and corrected for multiple comparisons controlling the false-discovery-rate (FDR) at *q* ≤ 0.05 (50) if not otherwise specified. Due to the large sample size rendering minor effects significant, results were also assessed considering effect size (*sr*^2^, *R*^2^, *d*, partial η^2^) and only deemed meaningful if there was at least a small effect (*sr*^2^ ≥ 0.02, *R*^2^ ≥ 0.01, *d* ≥ 0.2, partial η^2^ ≥ 0.01) according to Cohen (51). Data handling and statistical analyses were performed with SPSS 25.0 (Armonk, NY: IBM Corp.). Effect size calculations and power analyses were either performed with SPSS, GPower [3.1, HHU Düsseldorf (52)], or an online calculator (53).

#### Analysis of (Co-)Variance

Comparisons between participants affected by thyroid autoimmunity (HT and TPO_only_) and thyroid dysfunction (subclinical or overt hypothyroidism or hyperthyroidism) with controls as well as between TPO_only_ and HT participants were performed by analyses of (co-)variance (AN(C)OVA) regarding the dependent variables KINDL-R and SDQ-TD scores, z-standardized thyroid hormone levels (TSH, fT4, and fT3), TPO-AB titers, urinary iodine excretion, and z-standardized thyroid volume. Covariates known to affect KINDL-R and SDQ-TD scores (age, gender, social status, migration background, and residency in East or West Germany) have previously been identified (44, 54). Despite partially ambiguous evidence, we also considered covariates affecting thyroid hormone levels (use of levothyroxine and iodine), BMI (55, 56), smoking (57), vitamin D status (58), combined oral contraceptives (59), TPO-AB titers [iodine exposition (60)], urinary iodine excretion (iodine supplementation), and thyroid volume [BMI (47)] for analyses. Covariates were considered for ANCOVA testing if there was a significant correlation and at least a small effect (*r* > ± 0.1) (61). Correlation analyses were performed considering the scale of measure (interval scaled variables: Pearson correlation *r*; interval scaled and dichotomous variable: point-biserial correlation *r*_*pb*_*;* interval scaled and ordinal scaled variable: Kendall τ) as indicated (for detailed information on the statistics performed and the evaluation of assumptions made by the statistical procedures employed, please refer to the Supplementary Material).

#### Multiple Regression

The relationship between KINDL-R and SDQ-TD scores and z-standardized thyroid hormone levels (TSH, fT4, fT3), z-standardized thyroid volume, urinary iodine excretion, TPO-AB levels was assessed for each thyroid disease group by multiple regression combining a standard hierarchical approach with stepwise regression. Covariates identified earlier to affect either the dependent variable (KINDL-R or SDQ-TD scores) or the independent variables (z-standardized TSH, fT4, fT3, and thyroid volume, urinary iodine excretion as well as TPO-AB level) were entered as the first block of regressors and assessed via stepwise regression. Thereafter, the independent variables outlined above were entered as a second block of regressors. The combined variance accounted for in KINDL-R and SDQ-TD scores by the independent variables (thyroid parameters) was assessed by testing the change in *R*^2^ against zero.

#### Demographics and Sensitivity Analysis

Differences in the frequency of gender, the use of levothyroxine or iodine, previously physician-diagnosed thyroid disease, and irregular echogenicity on ultrasonography were assessed in comparison to healthy participants as well as between HT and TPO_only_ participants by χ2 tests of independence and Fisher's exact test in case of cell counts <5. Age and BMI were compared between groups by ANOVAs and *post-hoc* tests.

Sensitivity analyses were conducted to verify results. Considering thyroid autoimmunity, we repeated analyses with a TPO-AB cut-off level of 200 IU/ml. Analyses regarding SC_HYPO_ and HYPER_SC_ were also conducted considering only participants with a TSH level above and below 3 SDS (99.9 and 0.01 percentile), respectively.

## Results

### KINDL-R and SDQ—ANCOVA

Neither transformed KINDL-R scores (*p* = 0.114; see Table 1B and Table S2 for detailed statistics regarding the analysis of KINDL-R and SDQ-TD scores) nor transformed SDQ-TD scores (*p* = 0.308) did significantly differ between participants with thyroid autoimmunity and healthy controls considering the via correlation analysis identified covariates age [KINDL: *r*_(7, 295)_ = −0.354, *p* < 0.001; SDQ: *r*_(7, 396)_ = −0.170, *p* < 0.001] and social status [SDQ: *r*_(7, 229)_ = −0.132, *p* < 0.001; s. Table 3 for detailed results]. Thus, H_1_ (impaired HRQoL and increased risk of mental health problems in HT) was rejected. Power for detecting a small effect (*d* = 0.3) regarding a mean difference in either KINDL-R or SDQ-TD scores between HT participants and healthy controls was adequate (0.79), however insufficient for TPO_only_ participants (Table 2).

**Table 2:** Results of the power analysis.

	**Outcome variable**	**HT**	**TPO_**only**_**	**Subclinical hypothyroidism**	**Subclinical hyperthyroidism**	**Overt hypothyroidism**	**Overt hyperthyroidism**
*d* = 0.3	KINDL-R	0.79	0.74	>0.99	>0.99	0.38	0.46
	SDQ	0.79	0.73	>0.99	>0.99	0.38	0.46
*d* = 0.5	KINDL-R	0.99	0.98	>0.99	>0.99	0.72	0.84
	SDQ	0.99	0.99	>0.99	>0.99	0.72	0.83

*Power to detect a small (d = 0.3) and medium (d = 0.5) effect size in mean difference in comparison to controls regarding KINDL-R and SDQ total difficulties scores separately for participants with thyroid autoimmunity and thyroid dysfunction*.

In participants affected by thyroid dysfunction, age [KINDL: *r*_(7, 809)_ = −0.350, *p* < 0.001; SDQ: *r*_(7, 922)_ = 0.167, *p* < 0.001] and social status [SDQ: *r*_(7, 738)_ = −0.136, *p* < 0.001, respectively] were identified as significant covariates. There was a significant difference regarding transformed SDQ-TD scores (*p* = 0.029) but not transformed KINDL-R scores (*p* = 0.253) in comparison to healthy controls. *Post-hoc* contrasts revealed a significant difference between the small subsample of participants with HYPO_OVERT_ and healthy controls (see Table 3 for a summary of contrasts performed) even though SDQ-TD scores in HYPO_OVERT_ were still within the normal range (M: 11.43, normal range < 15). A likewise significant *post-hoc* contrast between participants with HYPER_SC_ and healthy controls, however with negligible effect size, did not survive the correction for multiple comparisons (*p* = 0.028, *d* = 0.14). In consequence, H_5_ (impaired HRQoL and increased risk of mental health problems in HYPO_overt_) and H_6_ (impaired HRQoL and increased risk of mental health problems in HYPER_overt_) could not be confirmed since there was no significant effect of overt thyroid dysfunction on HRQoL or mental health. H_3_ (unaffected HRQoL and mental health in HYPO_SC_), however, was supported as we did not observe any effect of HYPO_SC_ on HRQoL. While power for detecting a small effect (*d* = 0.3) regarding a difference in either KINDL-R or SDQ-TD scores between HYPO_SC_ and HYPER_SC_ and healthy controls was sufficient (0.99), power in participants affected by HYPO_OVERT_ and HYPER_OVERT_ was not (Table 2; for results of the sensitivity analysis, please see Supplementary Material).

**Table 3:** AN(C)OVA results.

	**HT**	**TPO_**only**_**	**Subclinical hypothyroidism**	**Subclinical hyperthyroidism**	**Overt hypothyroidism**	**Overt hyperthyroidism**
KINDL	−0.69 [−3.00; 1.61] *p* = 0.555	2.50 [0.04; 4.95] *p* = 0.046	−0.32 [−1.82; 1.20] *p* = 0.999	−0.13 [−1.80; 1.53] *p* = 0.999	4.27 [−1.70; 10.24] *p* = 0.371	1.76 [−3.38; 6.90] *p* = −984
SDQ	0.38 [−0.75; 1.52] *p* = 0.507	−0.86 [−2.07; 0.36] *p* = 0.169	0.19 [−0.35; 0.71] *p* = 0.484	−0.65 [−1.22; −0.07] *p* = 0.028, *d* = 0.14	**−2.40** ** [−4.45; −0.35]** ***p*** **= 0.022**, ***d*** **= 0.52**	0.29 [−2.05; −1.48] *p* = 0.751
z–TSH	**−1.37** ** [−1.57; −1.16]** ***p*** **< 0.001*****, d*** **= 1.17**	**−0.43** ** [−0.65; −0.2]** ***p*** **< 0.001**, ***d*** **= 0.51**	**−2.44** ** [−2.54; −2.35]** ***d*** **= 3.49**	**2.56** ** [2.46; 2.67]** ***d*** **= 3.33**	**−4.25** ** [−4.62; −3.89]** ***d*** **= 1.64**	**3.67** ** [3.35; 3.99]** ***d*** **= 2.08**
z-fT4	0.00 [−0.20; 0.21] *p* = 0.967	0.09 [−0.13; 0.31] *p* = 0.443	[−0.09; 0.10] *p* = 0.917	**−0.13** ** [−0.23; −0.02]** ***p*** **= 0.016*****, d*** **= 0.15**	**3.39** ** [3.01; 3.77]** ***p*** **< 0.001**, ***d*** **= 1.71**	**−3.26** ** [−3.58; −2.93]** ***p*** **< 0.001**, ***d*** **= 2.48**
z–fT3	0.08 [−0.16; 0.31] *p* = 0.527	−0.24 [−0.50; 0.01] *p* = 0.055	**−0.30** ** [−0.41; −0.18]** ***p*** **< 0.001*****, d*** **= 0.29**	**−0.33** ** [−0.45; −0.20]** ***p*** **< 0.001*****, d*** **= 0.24**	**0.95** ** [0.50; 1.40]** ***p*** **< 0.001*****, d*** **= 0.75**	**−2.16** ** [−2.54; −1.78]** ***p*** **< 0.001*****, d*** **= 0.99**
TPO-AB	**−390.81** ** [−453.43; −328.18]** ***p*** **< 0.001*****, d*** **= 1.45**	**−200.83** ** [−266.54; −135.11]** ***p*** **< 0.001**, ***d*** **= 1.51**	–	–	–	–
Urinary iodine excretion	−4.03 [−24.54; 16.48] *p* = 0.700	−1.41 [−22.89; 20.07] *p* = 0.898	**−16.26** ** [−25.46; −7.06]** ***p*** **< 0.001*****, d*** **= 0.19**	**−19.55** ** [−29.78; −9.33]** ***p*** **< 0.001*****, d*** **= 0.24**	−12.53 [−52.41; 27.34] *p* = 0.538	−17.94 [−49.49; 13.61] *p* = 0.265
z-thyroid volume^*^	**−2481.12** ** [−2968.43; −1993.82]** ***p*** **< 0.001**, ***d*** **= 1.26**	**−1535.63** ** [−2076.89; −994.16]** ***p*** **< 0.001**, ***d*** **= 0.84**	**452.59** ** [192.10; 751.31]** ***p*** **= 0.001*****, d*** **= 0.20**	−275.67 [−571.04; 9.71] *p* = 0.058	278.28 [−729.41; 1285.97] *p* = 0.588	**−1227.90** ** [−2083.37; −372.44]** ***p*** **= 0.005*****, d*** **= 0.62**

Significant contrasts are marked in bold, d refers to Cohen's effect size measure comparing means. Please note that differences in means may not correspond to results presented in Table 1B in case of ANCOVA testing and correction of means for covariates.

* *Results refer to differences in ranks but not z-standardized thyroid volume. Mean differences and the 95% confidence intervals in brackets between the respective group as outlined in each column and healthy controls FDR-corrected for multiple testing at q < 0.05*.

### KINDL-R and SDQ—Multiple Regression

Only in healthy controls but in neither group affected by thyroid dysfunction or autoimmunity, thyroid parameters (z-standardized TSH, fT4, and fT3 levels, z-standardized thyroid volume and urinary iodine excretion) accounted for a significant, however negligible, proportion of variance in KINDL-R scores (*R*^2^ = 0.002) in addition to age, social status, and BMI. Moreover, in neither group, thyroid parameters accounted for a significant proportion of variance in SDQ-TD scores when analyses were corrected for multiple comparisons (Table 4).

**Table 4:** Regression results.

	**Controls**		**HT**		**TPO_**only**_**		**Subclinical hypothyroidism**		**Subclinical hyperthyroidism**		**Overt hypothyroidism**		**Overt hyperthyroidism**	
	**KINDL**	**SDQ**	**KINDL**	**SDQ**	**KINDL**	**SDQ**	**KINDL**	**SDQ**	**KINDL**	**SDQ**	**KINDL**	**SDQ**	**KINDL**	**SDQ**
z-TSH	**0.030** ** [*****t***_**(6, 264)**_ **= 2.50]** ***p*** **= 0.013** ** (*****sr**^**2**^* **= 0.001)**	−0.025 [*t*_(6, 211)_ = −1.98] *p* = 0.047 (*sr^2^* = 0.001)	−0.040 [*t*_(60)_ = −0.27] *p* = 0.787	−0.072 [*t*_(60)_ = −0.51] *p* = 0.614	0.254 [*t*_(49)_ = 1.86] *p* = 0.069	−0.222 [*t*_(47)_ = −1.18]*p* = 0.122	0.010 [*t*_(247)_ = 0.15] *p* = 0.878	0.027 [*t*_(234)_ = 0.42] *p* = 0.677	0.150 [*t*_(187)_ = 2.14] *p* = 0.034 (*sr*^2^ = 0.02)	0.009 [*t*_(180)_ = 0.12] *p* = 0.906	0.449 [*t*_(13)_ = 0.70] *p* = 0.498	−0.755 [*t*_(13)_ = −1.37] *p* = 0.195	−0.315 [*t*_(19)_ = −0.97] *p* = 0.344	0.105 [*t*_(19)_ = 0.30] *p* = 0.770
z-fT4	−0.005 [*t*_(6, 264)_ = −0.42] *p* = 0.673	−0.011 [*t*_(6, 211)_ = −0.86] *p* = 0.390	−0.158 [*t*_(60)_ = −1.12] *p* = 0.268	0.231 [*t*_(60)_ = 1.67] *p* = 0.101	−0.01 [*t*_(49)_ = −0.06] *p* = 0.949	0.241 [*t*_(47)_ = 1.63] *p* = 0.110	0.045 [*t*_(247)_ = 0.70] *p* = 0.486	−0.082 [*t*_(234)_ = −1–24] *p* = 0.215	−0.009 [*t*_(187)_ = −0.13] *p* = 0.901	−0.070 [*t*_(180)_ = −0.95] *p* = 0.343	−0.24 [*t*_(13)_ = −0.34] *p* = 0.740	0.321 [*t*_(13)_ = 0.53] *p* = 0.605	−0.145 [*t*_(19)_ = −0.47] *p* = 0.646	0.193 [*t*_(19)_ = 0.55] *p* = 0.591
z-fT3	0.025 [*t*_(6, 264)_ = 2.04] *p* = 0.024 (*sr^2^* < 0.001)	−0.012 [*t*_(6, 211)_ = −0.98] *p* = 0.329	0.031 [*t*_(60)_ = 0.24] *p* = 0.811	0.170 [*t*_(60)_ = 1.32] *p* = 0.191	0.183 [*t*_(49)_ = 1.25] *p* = 0.216	−0.159 [*t*_(497)_ = −1.05] *p* = 0.298	0.056 [*t*_(247)_ = 0.87] *p* = 0.387	0.074 [*t*_(234)_ = 1.12] *p* = 0.266	−0.051 [*t*_(187)_ = −0.69] *p* = 0.490	0.119 [*t*_(180)_ = 1.52] *p* = 0.130	0.354 [*t*_(13)_ = 1.05] *p* = 0.314	−0.686 [*t*_(13)_ = −2.37] *p* = 0.034 (*sr^2^* = 0.02)	−0.206 [*t*_(19)_ = −0.56] *p* = 0.585	0.039 [*t*_(19)_ = 0.10] *p* = 0.925
TPO-AB	^#^	^#^	−0.096 [*t*_(65)_ = −0.72] *p* = 0.472	0.023 [*t*_(60)_ = 0.175] *p* = 0.861	−0.007 [*t*_(49)_ = −0.05] *p* = 0.959	−0.043 [*t*_(47)_ = −0.30] *p* = 0.765	^#^	^#^	^#^	^#^	^#^	^#^	^#^	^#^
Urine iodine	0.020 [*t*_(6, 264)_ = 1.66] *p* = 0.097	<0.001 [*t*_(6, 211)_ = 0.12] *p* = 0.991	^#^	^#^	^#^	^#^	0.024 [*t*_(247)_ = 0.39] *p* = 0.692	−0.060 [*t*_(234)_ = −0.94] *p* = 0.348	0.044 [*t*_(187)_ = 0.62] *p* = 0.537	−0.030 [*t*_(180)_ = −0.41] *p* = 0.685	^#^	^#^	^#^	^#^
z-thyroid volume	−0.010 [*t*_(6, 264)_ = −0.93] *p* = 0.408	−0.006 [*t*_(6, 211)_ = −0.47] *p* = 0.635	−0.123 [*t*_(65)_ = −0.88] *p* = 0.385	−0.095 [*t*_(60)_ = −0.69] *p* = 0.496	−0.166 [*t*_(49)_ = −1.18] *p* = 0.244	0.049 [*t*_(47)_ = 0.33] *p* = 0.740	−0.073 [*t*_(247)_ = −1.20] *p* = 0.230	0.068 [*t*_(234)_ = 1.08] *p* = 0.281	−0.070 [*t*_(187)_ = −1.00] *p* = 0.317	0.096 [*t*_(180)_ = 1.32] *p* = 0.187	0.363 [*t*_(13)_ = 1.62] *p* = 0.130	−0.027 [*t*_(13)_ = −0.14] *p* = 0.892	−0.180 [*t*_(19)_ = −0.70] *p* = 0.494	0.137 [*t*_(19)_ = 0.57] *p* = 0.577
R*^2^*${}_{\text{Thyroid}}^{*}$	**0.002** ** [*****F***_**(4, 6264)**_ **= 3.02]** ***p*** **= 0.01**	0.001 [*F*_(4, 6211)_ = 1.27] *p* = 0.276	0.053 [*F*_(5, 60)_ = 0.67] *p* = 0.651	0.023 [*F*_(5, 60)_ = 1.14] *p* = 0.348	0.131 [*F*_(5, 49)_ = 1.47] *p* = 0.215	0.131 [*F*_(5, 47)_ = 1.41] *p* = 0.237	0.013 [*F*_(5, 247)_ = 0.68] *p* = 0.639	0.017 [*F*_(5, 234)_ = 0.89] *p* = 0.491	0.031 [*F*_(5, 187)_ = 1.313] *p* = 0.260	0.019 [*F*_(5, 184)_ = 0.78] *p* = 0.568	0.374 [*F*_(4, 13)_ = 1.94] *p* = 0.164	0.541 [*F*_(4, 13)_ = 3.83] *p* = 0.029	0.057 [*F*_(4, 19)_ = 0.29] *p* = 0.882	0.046 [*F*_(4, 19)_ = 0.30] *p* = 0.919

*Significant findings FDR-corrected for multiple testing at q <0.05 are marked in bold, sr^2^ is the squared semipartial correlation*.

# *Indicates regressors not tested in the respective case for reasons outlined in the results section*.

* *Indicates the proportion of variance in KINDL and SDQ scores accounted for by all the above thyroid parameters. Standardized regression coefficients (β) from multiple regression separately for participants with thyroid autoimmunity and thyroid dysfunction with the dependent variable KINDL-R score and SDQ total difficulties score*.

Despite a significant but again negligible linear relationship (*sr*^2^ = 0.001) between z-standardized TSH levels and KINDL-R scores in healthy controls, no such association was detected between any single thyroid parameter and KINDL-R or SDQ-TD scores in any other group affected by thyroid dysfunction or autoimmunity when considering the confounding factors age, social status, and BMI and a correction for multiple comparisons. These findings are in line with H_4_ (no correlation between HRQoL and thyroid hormone status in HYPO_SC_) but result in rejection of H_2_ (negative correlation between TPO-AB levels and HRQoL in HT).

Also in healthy controls, z-standardized fT4, fT3, thyroid volume, and urinary iodine excretion did not account for a significant proportion of variance in KINDL-R or SDQ-TD scores when accounting for multiple testing and considering the covariates age, social status, and BMI.

Note, TPO-AB levels were only regressed on KINDL-R and SDQ-TD scores in participants with thyroid autoimmunity but not in participants with thyroid dysfunction as the number of participants with detectable TPO-AB levels in the latter case was insufficient for meaningful analyses. This also applied to urinary iodine excretion, which was excluded from regression analyses in participants with HYPO_OVERT_ and HYPER_OVERT_.

### TPO_only_

There was *no* significant difference between the TPO_only_ and the HT group regarding transformed KINDL-R (*p* = 0.062; see Table S3 for detailed statistics) or transformed SDQ-TD scores (*p* = 0.142).

Regarding thyroid parameters, both groups significantly differed with regard to z-standardized TSH (*p* < 0.001, *d* = 0.82; lower in TPO_only_), TPO-AB titers (*p* < 0.001, *d* = 0.27; lower in TPO_only_) as well as the frequency of a previously physician-diagnosed thyroid disease ($p_{Fisher^{\prime }s\;exact\;test}$ < 0.001, *d* = 2.14), the frequency of levothyroxine prescription $p_{Fisher^{\prime }s\;exact\;test}$ < 0.001, *d* = 1.86), and structural irregularities on thyroid ultrasonography $p_{Fisher^{\prime }s\;exact\;test}$ < 0.001, *d* = 1.11).

## Discussion

Studies on HRQoL and mental health in children and adolescents affected by thyroid disease are rare, and findings are ambiguous. Moreover, children and adolescents with subclinical thyroid dysfunction and subclinical thyroid autoimmunity have not yet been sufficiently characterized with regard to thyroid function parameters as well as demographic aspects. Thus, based on a nationwide cross-sectional study, we investigated the relationship between HRQoL as well as mental health on the one hand and thyroid dysfunction and autoimmunity on the other hand.

Contrary to our hypotheses which based on findings in adults, neither HRQoL nor mental health was significantly affected in children and adolescents with subclinical or overt thyroid dysfunction and with thyroid autoimmunity. The only exception were higher scores on the SDQ in HYPO_OVERT_ in comparison to healthy controls. However, these scores were nonetheless within the normal range of normative data (Tables 1B, 3). Moreover, in neither group affected by thyroid dysfunction or autoimmunity, thyroid hormone levels (TSH, fT4, fT3), TPO-AB levels, urinary iodine excretion, or thyroid volume accounted for a significant proportion of variance in KINDL-R or SDQ-TD scores nor did these variables show a significant (linear) relationship with HRQoL or mental health (Table 4).

### Hashimoto's Thyroiditis

Despite sufficient statistical power, HT did not have an effect on HRQoL or mental health in children and adolescents, which was confirmed by sensitivity analysis. Therefore, H_1_ and H_2_ were rejected.

In adults affected by HT, an impairment of HRQoL was demonstrated by a considerable number of studies (12–16). Remarkably, these impairments seem to persist in a relevant proportion of patients despite treatment with levothyroxine (1, 17). Reasons discussed to explain this finding have been summarized by Jonklaas (62) and include an elevated BMI in HT, disease awareness as well as comorbidities in patients with HT. Children and adolescents with HT in the present study, however, did not significantly differ from healthy controls regarding their BMI, had not been pre-diagnosed with an autoimmune affection of the thyroid gland in most of the cases and were in either good or even very good general health (Table 1A). Thus, the psychological effects of a diagnosis of chronic disease and misattribution of symptoms caused by comorbidities are unlikely. In addition to these considerations, Nexø et al. (63) studied experiences of patients with thyroid dysfunction by qualitative interviews and identified social support and acceptance as an important aspect of coping in HT. Indeed, while various aspects of HRQoL are unaffected, impairment of social functioning is a consistent finding in adults with HT [e.g., (1, 12, 15, 16)] and, therefore, could be one of the main drivers of compromised HRQoL. In children and adolescents, there was no evidence of adversely affected social functioning, as evidenced by exploratory subscale analyses of the KINDL-R and SDQ (data not shown). In summary, reasons discussed to explain an impaired HRQoL in adults with HT do not apply to children and adolescents, and this may explain why HRQoL is unaffected by HT in the latter.

Despite reasons concerning disease-related cognition and self-perception discussed above, autoimmunity itself may be the cause for impaired HRQoL and behavioral problems in HT in adults. Autoimmunity in HT may not only affect the thyroid gland but also act systemically (64) and thereby target the brain as summarized by Leyhe and Müssig (65). It has been shown that TPO-ABs bind to cerebellar astrocytes (66), a mechanism by which a direct effect of TPO-ABs on the brain is feasible. Moreover, TPO-ABs may only indicate an epiphenomenon of systemic autoimmunity as ABs directed against the central nervous system (CNS) have been found in a significant proportion of adult patients with HT. These CNS ABs disturb myelogenesis, induce inflammation, and thereby potentially impair neurotransmission and contribute to the clinical phenotype of HT, including an impaired HRQoL and mental health observed in adults (21). In children and adolescents, no study so far has investigated the prevalence of AB targeting the CNS. However, children and adolescents with HT are at an increased risk for multiple autoimmune affections (67–69) that may (or may not) become apparent over time on an undetermined scale (68, 70). Thus, there could be an increased risk for an autoimmune disease of the CNS in pediatric HT, which may prospectively impair HRQoL. Considering the present results and the hypothesized effect of CNS-AB on mental health, an autoimmune affection of the CNS does not seem to affect a significant proportion of children and adolescents examined in the present study.

### TPO_only_

In TPO_only_ participants, there was no effect of thyroid autoimmunity on HRQoL and mental health by the analysis of (co-)variance and multiple regression.

A rise in the prevalence of TPO-AB positivity has been described with the onset of puberty, especially in girls (24–26). Even though many children and adolescents affected by TPO-AB positivity do not develop overt thyroid dysfunction (26, 71), the same autoimmune mechanisms may be at work as in HT. Thus, we also investigated the relationship between HRQoL, mental health, and TPO-AB positivity in the absence of thyroid dysfunction. As in children affected by HT, there was no significant impairment of HRQoL or an increased risk of mental health problems, most likely for the reasons outlined above.

However, regarding thyroid function, TPO_only_ participants evidenced thyroid hormone levels, TPO-AB titers, and thyroid volume intermediary between participants affected by HT and healthy controls. While findings were still within the normal range, this may indicate harboring overt disease as in participants with HT. This is in line with conclusions derived by Prummel and Wiersinga (72) that increased TPO-AB titers pose affected individuals at risk for developing overt autoimmune disease. Concerning demographic variables, there was no significant difference between TPO_only_ and HT participants supporting the notion that they originate from the same population but may face autoimmunity with a different pace of progression. However, as already pointed out by Beastall (71), longitudinal studies are needed to gain further insights into this condition and to determine whether TPO-AB positivity in affected individuals is a transient phenomenon associated with puberty, a separate entity of thyroid affection in adolescence and early adulthood or just an early stage of HT. Moreover, the present study was underpowered to detect small effects on either outcome measure and, therefore, additional studies with an adequate sample size are needed.

### Subclinical Hypothyroidism

In line with H_3_ and H_4_, we did neither find an effect of HYPO_SC_ on HRQoL and mental health, nor did we observe a significant linear relationship between thyroid function parameter and our outcome measures.

Previously, Holtmann et al. (7) reported significantly higher TSH levels in children and adolescents with severe mood problems, which could not be confirmed by Zepf et al. (9), relying on the same questionnaire and a similar research methodology but a larger sample. However, only a small fraction of children and adolescents in either study was affected by HYPO_SC_ when applying a pediatric reference range for TSH. Drawing on a sample of children and adolescents affected by HYPO_SC_, Cerbone et al. (8) found no relationship between HYPO_SC_ and mental health, which is supported by results of the present study as well as the two most recent meta-analyses of the relationship between HYPO_SC_ and depression in adults (73, 74). Tang et al. (74) showed in a subgroup analysis that only adults aged 50 and above were more likely to experience depression which agrees with considerations by Dayan and Panicker (75), arguing that an underlying chronic condition, more likely to occur in the elderly, could be the cause of increased rates of depression in adults rather than HYPO_SC_. This suggestion is plausible since there is no known physiological mechanism by which HYPO_SC_ would cause significant effects on HRQoL and mental health in the presence of peripheral euthyroidism and without concurrent thyroid autoimmunity.

### Subclinical Hyperthyroidism

In the present study, we did not find a difference in participants affected by HYPER_SC_ and healthy controls with regard to HRQoL and mental health despite sufficient statistical power also to detect even small effects.

In adults, there is ambiguous evidence regarding the effect of HYPER_SC_ on HRQoL and mental health if at all investigated (27, 30–33). Unfortunately, most studies do not report subscale findings of the HRQoL measures employed. However, as can be concluded from the study conducted by Klaver et al. (33), there may be a slight impairment of HRQoL due to symptoms caused by HYPER_SC_, even though other areas of HRQoL were unaffected. In contrast to these findings in adults, most children and adolescents affected by HYPER_SC_ were not diagnosed with a thyroid disorder, which argues against significant clinical symptoms, either physical or mental. Moreover, 86% of children and adolescents were in either good or very good general health, and, as in HYPO_SC_, there is no plausible physiological mechanism by which HYPER_SC_ would act on HRQoL and mental health in the presence of peripheral euthyroidism and without concurrent thyroid autoimmunity. Also, note that a sensitivity analysis considering only participants of the survey with severely reduced TSH levels verified these results.

### Overt Hypothyroidism

As hypothesized (H_5_) and previously found in adults (21), also children and adolescents affected by HYPO_OVERT_ scored higher on the SDQ, indicating more mental health problems. However, scores were still well within the normal range of normative data (*M*: 11.42; normal range: <11 years: <16; >11 years: <17).

Using depression as a proxy for (severe) behavioral and emotional problems, different mechanisms have been proposed to explain the relationship between HYPO_OVERT_ and depression in adults. Among those mechanisms discussed, there is altered serotonin and catecholamine signaling, as well as a disturbed hypothalamic pituitary adrenal axis (76–78). For some of these mechanisms, the direction of effect and causality has not yet been conclusively established. The small effect of HYPO_OVERT_ on mental health found in the present study indicates that either these mechanisms are not at work in children and adolescents or that their impact on mental health is mitigated, likely due to moderating factors. As can be seen from Table 1A, a significant proportion of participants with HYPO_OVERT_ was affected by HT. Therefore, the same reasons as outlined above in the section related to findings in HT may not only explain why there was only a small effect of HYPO_OVERT_ on mental health in children and adolescents but also why there was no impact on HRQoL. This especially applies to the observation that most participants of the KiGGS study with HYPO_OVERT_ were in at least good general health, and only a small fraction was diagnosed with pre-existing thyroid disease. Thus, in most participants with HYPO_OVERT_, there was likely no disease awareness, which may in itself impair mental health to a significant extent (62).

However, the number of participants affected by HYPO_OVERT_ was small and, therefore, we argue for replication of findings with a sufficient sample size before any further conclusion can be drawn.

### Overt Hyperthyroidism

In adults, there is consistent evidence of a significantly impaired HQRoL with HYPER_OVERT_ (32, 37, 38). Moreover, only recently, Zader et al. (39) published evidence of a profoundly increased risk of a mental health disorder in children and adolescents affected with HYPER_OVERT_ based on the analysis of a large data repository coding previously physician-diagnosed medical conditions. However, in contrast to these findings and our hypotheses (H_6_), there was neither a reduced HQRoL nor an increased risk for mental health problems in the present study. These discrepancies might be related to the fact that Zader et al. (39) focused on children and adolescents seeking health care advice due to significant symptoms, which eventually resulted in physician-diagnosed thyroid dysfunction, while the present study focused on the general pediatric population. Differences between both study populations also become apparent when considering etiologically relevant aspects of the multifactorial relationship between hyperthyroidism and mental health problems suggested by Zader et al. (39). Zader et al. (39) argued that an overlap of symptoms associated with hyperthyroidism and mental health disorders, the detrimental effect of a diagnosis of a chronic disease or a biological process, namely autoimmunity, may explain their observation. However, about 90% of parents in the present study indicated good or even very good health in their children with HYPER_OVERT_, and the majority (about 80%) had not been diagnosed with thyroid disease (Table 1A). With regard to autoimmunity, the same considerations, as made above with regard to HT, may apply. The children and adolescents studied did not (yet) seem to be affected by systemic autoimmunity to a significant proportion.

The number of participants affected by HYPO_OVERT_ in the present study was small and, therefore, also the power to detect a significant effect with regard to HRQoL and mental health in HYPER_OVERT_ was insufficient. As in the case of HYPO_OVERT_, the present findings need to be replicated with an adequate sample size to detect an even small affection of HRQoL and mental health in these individuals.

### General Pediatric Population

In the general pediatric population without evidence of thyroid dysfunction, there was no significant relationship between HRQoL or behavioral problems and thyroid function parameters. While it has previously been shown that in the same sample, TSH levels within the reference range were associated with lipid levels (79) and blood pressure (80), there is no evidence of an association of either thyroid hormone parameter with HRQoL. Prior studies, however, did not account for multiple testing. Moreover, either no effect size measure was reported, or effects sizes were small or even negligible despite the large sample analyzed that favors statistical significance in the presence of irrelevant clinical effects. Thus, there is no conclusive evidence of a relationship between thyroid hormone levels and either physical or psychological functioning in children and adolescents from the KiGGS study.

### Limitations

The present study was cross-sectional by design and, therefore, causal inference is limited. Some authors argue that a diagnose of HYPO_SC_ and HYPER_SC_ should only be established by 2 blood samples with evidence of thyroid dysfunction taken on different occasions (5). Especially in epidemiological studies, however, this is not possible. Moreover, and as already outlined above, further longitudinal studies are needed to classify euthyroid TPO-AB positivity as either a transient phenomenon associated with puberty, a separate entity of thyroid affection in adolescence and early adulthood, or just an early stage of HT.

The number of participants with HT may have been underestimated as only TPO-AB but not Tg-AB were determined. Moreover, (ultrasound) investigators were only trained on performing a standardized volumetric evaluation of the thyroid gland but not to identify structural irregularities indicative of HT. This may also explain that in the present study the frequency of hypoechogenicity in HT was only about 60% the frequency previously reported, for example, by Kaloumenou et al. (25) in a likewise epidemiological study. However, this does not compromise the validity of results concerning HRQoL and mental health as findings in participants with thyroid autoimmunity were confirmed by sensitivity analysis, as discussed above.

## Conclusions

This is the first study to investigate the relationship between HRQoL, mental health, and thyroid (dys-) function and thyroid autoimmunity in children and adolescents in a large, nationwide study. Importantly, the study was sufficiently powered to detect even small effects of thyroid functioning on mental health. The conclusions we draw rely on stringent control of type I error, and findings are interpreted considering effect size and not mere statistical significance, which is inadequate due to the large sample size.

In contrast to adults, children and adolescents affected by thyroid disease and autoimmunity did not show significantly impaired HRQoL or mental health. These findings should, therefore, result in efforts to better understand the socio-psychological and (patho-)physiological differences between adults and children and adolescents. Thus, we believe our findings hold (prospective) value to basic research but also clinical care in dealing with children and adolescents as well as adults affected by thyroid disease.

## Data Availability Statement

The datasets for this article are not publicly available as the results reported are based on a secondary analysis of data provided by the Robert Koch Institute (RKI), Germany. Requests to access the datasets should, therefore, be directed to the RKI (kiggsinfo@rki.de).

## Ethics Statement

The KiGGS study was reviewed and approved by Charité Berlin ethics committee as well as the Federal Office for the Protection of Data. Written informed consent to participate in this study was provided by the participants' legal guardian/next of kin.

## Author Contributions

RH, AK, and CG conceptualized the study. RH analyzed and interpreted the data and wrote the manuscript. BH, AH, HH, and CG participated in scientific discussions and revised the manuscript. All authors contributed to the article and approved the submitted version.

## Conflict of Interest

The authors declare that the research was conducted in the absence of any commercial or financial relationships that could be construed as a potential conflict of interest.
