# Supplementary material for: No Effect of Thyroid Dysfunction and Autoimmunity on Health-Related Quality of Life and Mental Health in Children and Adolescents: Results From a Nationwide Cross-Sectional Study
Source: Front Endocrinol (Lausanne). 2020 Sep 2;11:454. doi: 10.3389/fendo.2020.00454 (PMC7492205; doi:10.3389/fendo.2020.00454)
Supplement: Supplementary file 1 [file Table_1.docx]

Supplementary Material

**Overview**

1. **Methods**
   1. Questionnaires
   2. z-Transformation of Thyroid Parameters
   3. Percentile Charts for Thyroid Volume
   4. Statistical Analyses: ANCOVA
   5. Statistical Analyses: Multiple Regression
2. **Results**
   1. Descriptives
   2. Thyroid Parameters
   3. Sensitivity Analysis
3. **References**
4. **Figures**
   1. S1 – Demographics: Detailed Statistics
   2. S2 – AN(C)OVA: Detailed Statistics
   3. S3 – Comparison Between TPO_only_ and HT: Detailed Statistics
   4. S4 – Sensitivity Analysis: Detailed Statistics

# Methods

## Questionnaires

Additional questionnaires administered to the parents covered information on the educational and professional status as well as on the total household income, which was used for calculating a social status score ranging from 3 to 21. Scores were computed for each parent separately, and the higher score was used to classify the child’s social status as lower, middle, or upper class (1).

## Transformation of Thyroid Parameters (TSH, fT4, fT3)

The transformation was performed by a two-step procedure using RefCurv (Version 0.4.4, <https://refcurv.com>; (2). First, a reverse computation algorithm, according to Fenton and Sauve (3), was employed to extract information on the distribution of TSH and fT4 from age- and gender-specific percentile charts published for the KiGGS survey participants (4). Second, data were z-transformed with respect to the extracted information.

## Percentile Charts for Thyroid Volume

For the construction of percentile curves for thyroid volume, only participants without a diagnosed thyroid disease, without thyroid medication (levothyroxine, iodine), without elevated TPO-AB levels above the assay cut-off and with normal thyroid hormone levels (TSH and fT4) were considered. Moreover, prior to the construction of reference curves, data was semi-winsorized. Semi-winsorizing refers to replacing outliers by a pre-defined value (5). Outliers were defined as thyroid volumes above and below 3 standard deviations (SDS) considering age and gender and replaced with a value corresponding to ± 3 standard SDS as part of the semi-winsorizing procedure outlined above. Thus, 0.5% of raw data was winsorized before analysis with RefCurv, which is well below a recommended threshold of 5% (5). RefCurv was used to create age- and gender-specific percentile curves for thyroid volume. RefCurv relies on the LMS method for the construction of percentile charts, as suggested by Cole (6). The LMS method assumes that a distribution of data can be normalized by Box-Cox transformation. The three parameters L (λ, skewness of distribution), M (μ, median) and S (σ, coefficient of variation) for Box-Cox transformation were selected by choosing the subset of hyperparameters providing the lowest Bayesian Information Criterion (BIC) after grid search considering 5 degrees of freedom for each hyperparameter (2). Model verification was performed by cross-validation as implemented in RefCurv. The best model fit (weighted against overfitting) was provided by 4 dfs in boys and 2 dfs in girls for the (penalized) splines of λ as well as 0 dfs for the (penalized) splines of μ and σ in boys as well as in girls.

## Statistical Analyses: ANCOVA

Normality of variables was assessed by the Kolmogorov-Smirnov test and visual inspection. Equality of variances was tested using residuals and Levene's test. Homogeneity of regression was investigated by separate ANCOVAs testing for the significance of interactions between the independent variable (grouping variable) and considered covariates (7). For specific tests, non-normally distributed variables were rank-transformed according to Templeton (8), preserving their mean and standard deviation. In the presence of heteroscedasticity and planed analysis of variance, we performed a robust ANOVA according to Welch (9). In the case of analysis of covariance and heterogeneity of regression as well as either heteroscedasticity or non-normally distributed residuals or both, we employed a non-parametric ANCOVA according to Quade (25) that is based on the comparison of residuals of a rank regression between dependent variable and covariate.

## Statistical Analyses: Multiple Regression

Normality of the dependent variables and residuals was assessed by the Kolmogorov-Smirnov test, homoscedasticity by the (modified) Breusch-Pagan test. The Durbin-Watson test excluded auto-correlation of residuals.

# Results

## Descriptives

Altogether, there were 68 **participants with HT**. Participants with HT were primarily female (77.9%, *p* < .001, *d* = 0.74) and on average older (*p* < .001, *d* = 0.73) than healthy controls, which is in line with hypothesis H_7_ (for detailed statistics see Table 1A, Table 1B and Table S1). About 1/5 of participants with HT were on medication with levothyroxine (*p*_Fisher’s exact test_ < .001, *d* = 4.51) and 9% were supplemented with iodine (*p*_Fisher’s exact test_ < .001, *d* = 4.04). 1/3 of HT participants had a physician-diagnosed thyroid disease (*p*_Fisher’s exact test_ < .001, *d* = 4.81) and presented with irregularities on thyroid ultrasound, (*p*_Fisher’s exact test_ < .001, *d* = 4.96). In addition, in 5 participants with HT a single thyroid nodule and in 1 participant a cyst were identified on ultrasonography. The most common diagnoses derived from ATC code were hypothyroidism (n = 7), Hashimoto’s thyroiditis (n = 4), and goiter (n = 5). In 54% of all HT cases 2, in 28% 3, in 13% 4, in 3% 5 and in 1,5% (n = 1) 6 of the criteria outlined in ‘Methods’ were fulfilled.

**Participants with TPO_only_** were on average older (*p* < .001, *d* = 0.47) and more often female (72.9%, *p* < .001, *d* = 0.59) than healthy controls. Among irregularities of the thyroid gland, there was 1 participant identified with a single cyst and 3 participants with single nodular lesions.

**Participants with HYPO_SC_** were on average younger (*p* < .001, *d* = 0.30), evidenced a higher frequency of TPO-positivity (*p*_Fisher’s exact test_ < .001, *d* = 3.90), were prescribed levothyroxine (*p*_Fisher’s exact test_ < .001, *d* = 3.03) as well as iodine more often (*p*_Fisher’s exact test_ < .001, *d* = 3.21) and were more frequently diagnosed with a thyroid disease (*p*_Fisher’s exact test_ < .001, *d* = 3.29) than healthy controls.

**Participants affected by HYPER_SC_** were on average younger (*p* < .001, *d* = 0.31), more often TPO-AB positive (*p*_Fisher’s exact test_ < .001, *d* = 2.97) and more often diagnosed with a thyroid disease (*p*_Fisher’s exact test_ = .001, *d* = 3.25) than healthy controls. One participant with HYPER_SC_ was treated with levothyroxine as well as iodine due to hypothyroidism.

**Participants with HYPO_OVERT_** were on average the youngest to be affected by a thyroid disorder. In 27.8% of participants affected by HYPO_OVERT_ we found positive TPO-AB levels (*p*_Fisher’s exact test_ < .001, *d* = 4.79) and 5% (n=1) were diagnosed with Graves’s disease and treated with propylthiouracil. A pattern of echogenicity typical for HT was found in about 5%.

**Participants with HYPER_OVERT_** were more often TPO-AB positive (*p*_Fisher’s exact test_ < .001, *d* = 4.29), were prescribed levothyroxine (*p*_Fisher’s exact test_ < .001, *d* = 4.35) as well as iodine more often (*p*_Fisher’s exact test_ = .004, *d* = 3.68), and were more often diagnosed with a thyroid disease (*p*_Fisher’s exact test_ = .031, *d* = 4.60) than healthy controls.

## Thyroid parameters

None of the considered covariates related significantly to z-standardized TSH, fT4 or fT3 levels, urinary iodine concentration, or TPO-AB titers. Smoking, vitamin D status and the use of oral contraceptives (COC) were discarded from analyses due to either a large number of missing values (vitamin D status (28.3-42.4%), smoking (4.4-54.7%)) or minor importance (COC usage only by 3.6% of girls affected by a thyroid disorder). Detailed descriptive and statistical results regarding thyroid function and parameters (z-standardized TSH, fT4, fT3, thyroid volume as well as TPO-AB levels and urinary iodine excretion) within each subgroup in comparison to healthy controls are summarized in Table 3 as well as Table S2.

## Sensitivity analysis

Considering only participants with a TPO-AB titer of at least 200 IU/ml, previous results were confirmed. There was no significant difference between participants affected by thyroid autoimmunity and healthy controls regarding either KINDL-R scores (HT: *M* = 73.23, *SD* = 8.72; HT_only_: *M* = 70.58, *SD* = 8.30; *p* = .379; s. Table S2 for detailed statistics) or SDQ-TD scores (HT: *M* = 9.84, *SD* = 3.97; HT_only_: *M* = 10.48, *SD* = 4.15; *p* = .677) as observed for a TPO-AB threshold of 100 IU/ml. Moreover, in neither group affected by thyroid autoimmunity, there was a significant relationship between thyroid parameters (z-standardized TSH, fT4, fT3 or thyroid volume, TPO-AB levels) and KINDL-R or SDQ-TD scores (s. Table S4) or significant variance accounted for by thyroid parameters as assessed by a change in R^2^ that survived the correction for multiple comparisons.

Analyses were also verified with respect to participants affected by HYPO_SC_ and HYPER_SC_ only considering cases with a z-standardized TSH above and below 3 SDS, respectively. Groups did not differ significantly from healthy controls regarding KINDL-R scores (HYPO_SC_: *M* = 74.78, _SD_ = 10.83; HYPER_SC_: *M* = 75.54, *SD* = 11.16; *p* = .230) and SDQ TD scores (HYPO_SC_: *M* = 10.30, *SD* = 4.73; HYPER_SC_: *M* = 9.30, *SD* = 5.08; *p* = .455) as previously observed. There was no significant relationship as identified by multiple regression between any thyroid parameter (z-standardized TSH, fT4, fT3 or thyroid volume and urinary iodine excretion) and KINDL-R or SDQ-TD scores.

# References

1. Kurth BM, Kamtsiuris P, Holling H, Schlaud M, Dolle R, Ellert U, et al. The challenge of comprehensively mapping children's health in a nation-wide health survey: design of the German KiGGS-Study. *BMC Public Health* (2008) 8:196. Epub 2008/06/06. doi: 10.1186/1471-2458-8-196. PubMed PMID: 18533019; PubMed Central PMCID: PMCPMC2442072.

2. Winkler C, Linden K, Mayr A, Schultz T, Welchowski T, Breuer J, et al. RefCurv: A Software for the Construction of Pediatric Reference Curves. *arXiv preprint arXiv:190109775* (2019).

3. Fenton TR, Sauve RS. Using the LMS method to calculate z-scores for the Fenton preterm infant growth chart. *Eur J Clin Nutr* (2007) 61(12):1380-5. Epub 2007/02/15. doi: 10.1038/sj.ejcn.1602667. PubMed PMID: 17299469.

4. Dortschy R, Rosario AS, Scheidt-Nave C, Thierfelder W, Thamm M, Gutsche J, et al. Bevölkerungsbezogene Verteilungswerte ausgewählter Laborparameter aus der Studie zur Gesundheit von Kindern und Jugendlichen in Deutschland (KiGGS). (2009).

5. Salkind NJ. *Encyclopedia of research design*: Sage (2010).

6. Cole TJ. The LMS method for constructing normalized growth standards. *Eur J Clin Nutr* (1990) 44(1):45-60. Epub 1990/01/01. PubMed PMID: 2354692.

7. Tabachnick BG, Fidell LS, Ullman JB. *Using multivariate statistics*: Pearson Boston, MA (2007).

8. Templeton GF. A two-step approach for transforming continuous variables to normal: implications and recommendations for IS research. *Communications of the Association for Information Systems* (2011) 28(1):4.

9. Delacre M, Leys C, Mora YL, Lakens D. Taking Parametric Assumptions Seriously: Arguments for the Use of Welch’s F-test instead of the Classical F-test in One-Way ANOVA. *International Review of Social Psychology* (2019) 32(1).

|  | | HT (*N* = 68) | | TPO_only_ (*N* = 59) | subclinical hypothyroidism  (*N* = 331) | | | subclinical hyperthyroidism (*N* = 2876) | overt hypothyroidism (*N* = 20) | | | overt hyperthyroidism (*N* = 28) |  |  |
| --- | --- | --- | --- | --- | --- | --- | --- | --- | --- | --- | --- | --- | --- | --- |
| age | | -2.00 [-2.81; -1.21] *p* < .001, *d* = 0.73 | | -1.58 [-2.43; -0.72] *p* < .001, *d* = 0.47 | 1.14 [0.76; 1.51] *p* < .001, *d* = 0.30 | | | 1.15 [0.74; 1.56] *p* < .001, *d* = 0.31 | 1.61 [0.11; 3.12] *p* = .035 | | | 0.01 [-1.28; 1.26] *p* = .989 |  |  |
| z-BMI | | -0.15 [-0.38; 0.09] *p* = .218 | | -0.20 [-0.45; 0.06] *p* = .135 | -0.14 [-0.30; -0.01] *p* = .097 | | | 0.08 [-0.09; 0.25] *p* = .887 | -0.14 [-0.76; 0.48] *p* = .999 | | | 0.29 [-0.24; 0.81] *p* = .741 |  |  |
| gender (female) | | χ2 (1, *N* = 7361) = 23.98 *p* < .001, *d* = 0.74 | | χ2 (1, *N* = 7352) = 14.36 *p* < .001, *d* = 0.59 | χ2 (1, *N* = 7293) = 0.460 *p* = .498 | | | χ2 (1, *N* = 7569) = 0.00 *p* = .984 | χ2 (1, *N* = 7313) = .078 *p* = .780 | | | χ2 (1, *N* = 7321) = 1.78 p = 0.183 |  |  |
| TPO-AB positivity | | 100% by definition | | 100% by definition | FET  *p* < .001, *d* = 3.90 | | | FET  *p* < .001, *d* = 2.97 | FET  *p* < .001, *d* = 4.79 | | | FET  *p* < .001, *d* = 4.29 |  |  |
| levothyroxine | | FET *p* < .001, *d* = 4.51 | | 0% by definition | FET  *p* < .001, *d* = 3.03 | | | FET  *p* = .036, *d* = 2.41 | 0% | | | FET  *p* < .001, *d* = 4.35 |  |  |
| iodine | | FET *p* < .001, *d* = 4.04 | | 0% by definition | FET  *p* < .001, *d* = 3.21 | | | FET  *p* = .036, *d* = 2.41 | 0% | | | FET  *p* < .001, *d* = 3.68 |  |  |
| diagnosed thyroid disease | | FET *p* < .001, *d* = 4.81 | | 0% by definition | FET  *p* < .001, *d* = 3.29 | | | FET  *p* < .001, *d* = 3.25 | FET  *p =* .003, *d* = 3.87 | | | FET  *p* < .001, *d* = 4.60 |  |  |
| altered echogenicity | | descriptive analysis | | 0% by definition | not tested | | | not tested | not tested | | | not tested |  |  |
| irregularities US | χ2 (1, *N* = 7361) = 2582.42 *p* < .001, *d* = 4.96 | | FET *p* < .001, *d* = 3.90 | | | FET  *p* < .001, *d* = 3.28 | FET  *p* < .001, *d* = 3.02 | | | FET  *p* < .001, *d* = 4.40 | FET  *p* < .001, *d* = 3.98 | | | |
| **Table S1**. Mean difference and 95% confidence interval brackets in case of age (years) and BMI or the χ2 test statistics with the degrees of freedom and the number of cases considered for testing in brackets comparing the respective group in each column with healthy controls. Significant effects are marked in bold, *d* refers to Cohen`s effect size measure. All cells related to results reported in the manuscript relying on Fisher`s exact test (FET) remain empty as SPSS does not provide a test statistic for this test. | | | | | | | | | | | | | |  |

|  | **standard analysis** | | **sensitivity analysis** | |
| --- | --- | --- | --- | --- |
|  | thyroid autoimmunity | thyroid dysfunction | thyroid autoimmunity | thyroid dysfunction |
| KINDL-R | *F*(2, 7291) = 2.17 *p* = .114 | *F*(4, 7803) = 1.34 *p* = .253 | *F*(2, 7241) = 0.97 *p* = .379 | *F*(2, 7248) = 1.47 *p* = .230 |
| SDQ | *F*(2, 7224) = 1.18 *p* = .308 | *F*(2, 7731) = 2.69 *p = .029* | *F*(2, 7174) = 0.39 *p* = .677 | *F*(2, 7183) = 0.79 *p* = .455 |
| z-TSH | *F*(2, 83.95) = 38.61* *p* < .001 | *F*(4, 83.98) = 2970.32** p <* .001 | # | # |
| z-fT4 | *F*(2, 83.82) = 0.26** p =* .769 | *F*(4, 83.53) = 35.71** p <* .001 | # | # |
| z-fT3 | *F*(2, 7414) = 2.05 *p =* .129 | *F*(4, 83.42) = 47.59** p* < .001 | # | # |
| TPO-AB | *F*(2, 83.17) = 68.81* *p* < .001 | # | # | # |
| urinary iodine excretion | *F*(2, 6498) = 0.08 *p* = .921 | *F*(4, 65.76) = 5.16** p =* .001 | # | # |
| z-thyroid volume | *F*(2, 7391) = 64.81^§^ *p* < .001 | *F*(4, 7819) = 5.99^§^ *p* < .001 | # | # |
| **Table S2**. Results of the AN(C)OVA analysis for all participants fulfilling the criteria of thyroid autoimmunity or thyroid dysfunction (standard analysis) as well as the sensitivity analysis only considering participants with a TPO-AB titer > 200 U/ml (thyroid autoimmunity) or a z-standardized TSH above and below 3 SDS (thyroid dysfunction). * Welch ANOVA due to heterogeneity of variances, ^§^ NP-ANCOVA, # not tested. | | | | |

|  | | | TPO_only_ | | |  |  |  |  |  |  |
| --- | --- | --- | --- | --- | --- | --- | --- | --- | --- | --- | --- |
| KINDL-R | | | 3.19 [-0.15; 6.54]  *p* = .062 | | |  |  |  |  |  |  |
| SDQ-TD | | | -1.24 [-2.89; 0.42]  *p* = .142 | | |  |  |  |  |  |  |
| z-TSH | | | 0.94 [0.64; 1.24]  *p* < .001* | | |  |  |  |  |  |  |
| z-fT4 | | | 0.08 [-0.22; 0.38]  *p* = .593 | | |  |  |  |  |  |  |
| z-fT3 | | | -0.32 [-0.66; 0.02]  *p* = .066 | | |  |  |  |  |  |  |
| TPO-AB | | | 189.98 [115.15; 264.81]  *p* < .001* | | |  |  |  |  |  |  |
| urinary iodine excretion | | | 2.61 [-26.96; 32.19]  *p* = .862 | | |  |  |  |  |  |  |
| z-thyroid volume | | | 945.60 [220.24; 1670.95]  *p* = .011* | | |  |  |  |  |  |  |
| Age | | | 0.43 [-0.73; 1.60] | | |  |  |  |  |  |  |
| z-BMI | | | -0.05 [-0.39; 0.30] | | |  |  |  |  |  |  |
| gender (female) | | | χ^2^(1, *N* = 131) = 0.438  *p* = .508 | | |  |  |  |  |  |  |
| TPO-AB positivity | | | 100% by definition | | |  |  |  |  |  |  |
| levothyroxine | | | FET* | | |  |  |  |  |  |  |
| iodine | | | FET  *p* = .030 | | |  |  |  |  |  |  |
| diagnosed thyroid disease | | | FET* | | |  |  |  |  |  |  |
| altered echogenicity | | | not tested | | |  |  |  |  |  |  |
| irregularities US | | | FET* | | |  |  |  |  |  |  |
| **Table S3.** Comparison of TPO_only_ participants with participants affected by HT. For an explanation of figures and abbreviations, please refer to Table S1 and S2. When not reported in the text, *p*-values are reported in the table. * indicates significant differences. | | | | | |  |  |  |  |  |  |
|  | | HT (*N* = 46) |  | | TPO_only_ (*N* = 25) |  | | subclinical hypothyroidism  (*N* = 29) |  | subclinical hyperthyroidism  (*N* = 52) |  |
|  | | KINDL-R | SDQ | | KINDL-R | SDQ | | KINDL-R | SDQ | KINDL-R | SDQ |
| z-TSH | | -.039 [t(41) = -0.22] *p* = .829 | .030 [t(41) = 0.18] *p* = .858 | | .357  [t(20) = 1.76] *p* = .094 | -.222 [t(19) = -1.04] *p* = .310 | | .203 [t(19) = 0.97] *p* = .343 | -.108 [t(18) = -0.45] *p* = .660 | .319 [t(31) = 2.04] *p* = .050 | -.094 [t(32) = -0.56] *p* = .582 |
| z-fT4 | | -.345 [t(41) = -2.04] *p* = .048 | .380 [t(41) = 2.39] *p* = .022 | | -.294 [t(20) = -1.46] *p* = .159 | .281 [t(19) = 1.32] *p* = .204 | | .381 [t(19) = 1.49] *p* = .152 | -.057 [t(18) = -0.19] *p* = .851 | .103 [t(31) = 0.64] *p* = .528 | -.232 [t(32) = -1.32] *p* = .197 |
| z-fT3 | | .173 [t(41) = 1.10] *p* = .280 | .211 [t(41) = 1.43] *p* = .161 | | -.219 [t(20) = -1.09] *p* = .480 | .086 [t(19) = 0.37] p = .714 | | - .118 [t(19) = -0.47] *p* = .642 | .214 [t(18) = 0.73] *p* = .478 | -.240 [t(31) = -1.32] *p* = .196 | .296 [t(32) = 1.43] *p* = .161 |
| TPO-AB | | - .020 [t(41) = -0.13] *p* = .896 | -.185 [t(41) = -1.23] *p* = .225 | | -.141 [t(20) = -0.72] *p* = .480 | .053 [t(19) = 0.24] *p* = .812 | | # | # | # | # |
| urinary iodine  excretion | | # | # | | # | # | | -.321 [t(19) = -1.47] *p* = .159 | .122 [t(18) = 0.47] *p* = .645 | .034 [t(31) = 0.22] *p* = .830 | .082 [t(32) = 0.45] *p* = .654 |
| z-thyroid volume | | -.152 [t(41) = -0.96] *p* = .341 | .315 [t(41) = 2.05] *p* = .046 | | -.036  [t(20) = -0.16] *p* = .875 | .178 [t(19) = 0.67] *p* = .510 | | -.158 [t(19) = -0.82] *p* = .422 | .025 [t(18) = 0.11]  *p* = .916 | -.428 [t(31) = -2.57] *p* = .015  (*sr^2^* = .14) | .303 [t(32) = 1.59] *p* = .122 |
| *R^2^*_Thyroid_* | | .167  [F(5, 41) = 1.647] *p* = .169 | .250 [F(5, 41) = 2.734] *p* = .032 | | .326 [F(5, 20) = 1.94] *p* = .133 | .273 [F(5, 19) = 1.25] *p* = .261 | | .155 [F(5, 19) = 0.90] *p* = .500 | .060 [F(5, 18) = 0.23] *p* = .945 | .223 [F(5, 31) = 2.16]  *p* = .085 | .13 [F(5, 32) = 0.95] *p* = .461 |

**Table S4**. Results of the sensitivity analysis only considering participants with a TPO-AB titer > 200 U/ml or a z-standardized TSH above and below 3 SDS. Standardized regression coefficients (β) from multiple regressions separately for participants with thyroid autoimmunity and thyroid dysfunction with the dependent variable KINDL-R score and SDQ-TD score. # indicates regressors not tested in the respective case for reasons outlined in the results section. * indicates the proportion of variance in KINDL-R and SDQ-TD scores accounted for by all the above thyroid parameters.
